# Differences in Oropharyngeal Viromes Between Surviving and Deceased Malayan Pangolins in a Rescue Context

**DOI:** 10.3390/microorganisms14092043

**Published:** 2026-09-13

**Authors:** Yi Min, Yanli Zhong, Tingting Jiang, Xiao Wei, Xia Meng, Deqiu Li, Peng Zeng, Meihong He, Chuntao Wei, Jianbao Wu, Yuxuan Qin, Diqi Yang, Tingting Yu

**Affiliations:** 1 School of Animal Science and Veterinary Medicine, Hainan University, Sanya 572025, China; 2 Terrestrial Wildlife Rescue and Epidemic Diseases Surveillance Center of Guangxi, Nanning 530025, China; 3 School of Tropical Agriculture and Forestry, Hainan University, Haikou 570228, China

**Keywords:** pangolin, wildlife rescue, pathology, metatranscriptomics, virome

## Abstract

Pangolins are heavily affected by illegal wildlife trade, but little is known about their viromes during rescue and recovery or their association with survival. Here, oropharyngeal swabs from rescued Malayan pangolins (*Manis javanica*) were examined by metatranscriptomic sequencing and compared between surviving (S1–S4) and deceased (D1–D4) individuals. Major organs from deceased pangolins were further examined histologically. The oropharyngeal virome was dominated by bacteriophages and arthropod- or environment-associated viral signals, but viral communities differed between outcome groups. Deceased pangolins showed higher alpha diversity than surviving pangolins, and beta-diversity analysis showed significant separation between groups. Phylogenetic analyses identified pestivirus-like, gammaretrovirus-like and picorna-like sequences that were detected mainly in deceased individuals, whereas Iflavirus- and Ghabrivirales-related sequences were most consistent with dietary or environmental exposure. Histopathology revealed multi-organ lesions in deceased pangolins, including interstitial lung injury, renal tubular degeneration and necrosis, hepatocellular steatosis and necrosis, tracheal mucosal injury, intestinal lesions and focal myocardial damage. These findings demonstrate heterogeneous oropharyngeal viral communities among rescued pangolins and reveal differences in viral community diversity and composition between surviving and deceased individuals. Given the small sample size and unequal sampling times between groups, these findings should be considered preliminary and exploratory and should not be interpreted as evidence of a causal relationship between viral signals and mortality. The study highlights the value of virome-based surveillance and careful discrimination between host-associated and exogenous viral signals in wildlife rescue settings.

## 1. Introduction

Virus transmission at the wildlife–livestock–human interface remains a central concern in the post-pandemic era, because host jumps are shaped by ecological contact, viral traits and host susceptibility [1,2]. Pangolins are among the wild mammals most affected by illegal trade, and confiscated animals commonly experience capture, crowding, transport, starvation and dehydration before rescue [3]. In addition to these extrinsic pressures, pangolin genomes show distinctive immune features, including pseudogenization of interferon epsilon (IFNE), a gene involved in epithelial innate defense in many eutherian mammals [4]. These ecological and host factors make rescued pangolins important subjects for studying viral exposure, animal health and potential spillover risk.

Since 2020, pangolin-associated virus research has expanded rapidly. Early studies focused mainly on SARS-CoV-2-related coronaviruses detected in Malayan pangolins [5,6,7,8,9,10,11], whereas later work used metagenomic or metatranscriptomic approaches to characterize broader pangolin-associated microbial and viral diversity [12,13,14,15]. Viruses reported from pangolins include coronaviruses, parainfluenza virus 5, pestiviruses, canine parvoviruses and other vertebrate- or invertebrate-associated viral signals [7,15,16,17,18,19]. However, many available datasets were generated from deceased animals, specific tissues or limited viral targets. Consequently, the relationship between viral community structure during rescue and subsequent survival remains poorly understood.

In this study, we analyzed the oropharyngeal virome of rescued Malayan pangolins and compared animals who survived with those that died during rescue. We further examined major organs from deceased individuals to document pathological lesions. By integrating metatranscriptomic profiling, phylogenetic analysis and histopathology, we aimed to characterize virome differences between rescued pangolins with different subsequent outcomes and to distinguish candidate host-associated viral signals from dietary or environmental signals.

## 2. Materials and Methods

### 2.1. Sample Collection

The classification of animals by final rescue outcome was used only for comparative virome analysis and did not imply that viral signals detected at admission were the sole cause of death. Available metadata, including sample identity, outcome group and sample type, are summarized in Table 1. Because the animals were legally confiscated and could not be experimentally manipulated, this study was designed as an observational analysis of rescue-associated viral profiles rather than an infection experiment. The outcome groups were therefore descriptive groupings rather than experimentally controlled infection or health-status groups.

All pangolins examined in this study were Malayan pangolins (*Manis javanica*) intercepted by Chinese customs and transferred to the Guangxi Zhuang Autonomous Region Terrestrial Wildlife Rescue, Research and Epidemic Disease Monitoring Center. According to rescue outcome, animals were assigned to a survival group (S1–S4) or deceased group (D1–D4). After admission and clinical stabilization, oropharyngeal swabs were collected with sterile swabs, placed in RNase-free cryovials containing viral preservation buffer, transported on liquid nitrogen or dry ice and stored at −80 °C until RNA extraction.

### 2.2. Tissue Sampling and Histopathology

Aseptic necropsies were performed on pangolins that died during rescue. Heart, liver, lung, spleen, kidney, trachea and intestinal tissues were collected. Representative tissue blocks were fixed in 10% neutral buffered formalin for 48 h, dehydrated through graded ethanol, cleared in xylene, embedded in paraffin and sectioned at 4–5 micrometers. Sections were stained with hematoxylin and eosin and examined by light microscopy. Remaining tissue aliquots were stored under RNase-free conditions for potential nucleic acid-based validation.

### 2.3. RNA Extraction, Library Preparation and Sequencing

No separate rRNA depletion step was performed before library preparation. Total RNA was extracted from oropharyngeal swab samples using TRIzol reagent (DP424; TIANGEN, Beijing, China), according to the manufacturer’s instructions. RNA quality and quantity were assessed prior to library preparation. RNA sequencing libraries were prepared using the NEBNext^®^ Ultra™ II RNA Library Prep Kit for Illumina^®^ (NEB #E7775L; New England Biolabs, Inc., Ipswich, MA, USA) following the manufacturer’s recommended procedures. The prepared libraries were sequenced on an Illumina NovaSeq 6000 platform (Illumina, Inc., San Diego, CA, USA) using a paired-end 150-bp (PE150) sequencing strategy. Approximately 6 Gb of sequencing data were generated per sample. Across the eight samples, 330,613,818 raw reads corresponding to 49.59 Gb of raw sequence data were obtained. Following quality filtering, 318,439,724 clean reads corresponding to 47.78 Gb of clean sequence data were retained. Host-derived sequences were subsequently removed, resulting in 313,896,204 non-host reads corresponding to 47.09 Gb of sequence data for downstream viral sequence analysis. Detailed sequencing statistics are provided in Table 2.

Raw reads, clean reads and non-host reads represent the numbers of sequencing reads before quality filtering, after quality filtering, and after removal of host-derived sequences, respectively. Raw bases, clean bases and non-host bases represent the corresponding sequencing data yields in gigabases (Gb). The error rate represents the sequencing error rate reported by the sequencing data quality-control pipeline. S1–S4 and D1–D4 denote the individual sample identifiers used throughout the study.

### 2.4. Virus Discovery and Abundance Estimation

This multi-step strategy was chosen to reduce overinterpretation of short or low-confidence viral matches. Database-supported viral hits were retained when they met the identity threshold, whereas divergent candidates were only considered further when conserved viral domains or prediction-based evidence supported viral origin. For interpretation, vertebrate-associated viruses, bacteriophages and likely dietary or environmental viruses were evaluated separately rather than pooled into a single pathogenic category.

Raw reads were processed with fastp to remove adapter-containing pairs, low-quality pairs in which more than 50% of bases had a Phred quality score of 5 or lower and reads containing more than 10% ambiguous bases. Host reads were removed with Bowtie2 using the parameters --end-to-end, --sensitive, -I 200 and -X 400. Clean non-host reads were assembled with Trinity (v2.15.0). Assembled sequences were queried against GenBank, NR and RefSeq virus databases using DIAMOND (version v2.1.24), and sequences showing at least 90% identity to viral references were classified as known viral sequences [12,15,20]. The remaining sequences were screened against the Conserved Domain Database for RNA-dependent RNA polymerase (RdRp) domains, and candidates not recovered by database searches were further assessed with DeepVirFinder (version 1.0) using a *p*-value threshold of 0.005. Viral abundance was calculated as fragments per kilobase of transcript per million mapped reads (FPKM). However, due to the fragmented nature of metatranscriptomic data, all assignments, particularly those based solely on partial RdRp regions, are considered preliminary and were cross-referenced with conserved domain and DeepVirFinder predictions to reduce false positives.

### 2.5. Diversity and Phylogenetic Analyses

Viral taxonomic labels were extracted from database annotations to generate relative abundance tables. Alpha diversity was measured using the Shannon index, and group differences were tested with the Wilcoxon rank-sum test [21]. Beta diversity was calculated from Bray–Curtis distances and visualized using principal coordinate analysis (PCoA), and group separation was evaluated by PERMANOVA (version 0.2.0). For selected viral lineages, open reading frames were predicted with ORFfinder (https://www.ncbi.nlm.nih.gov/orffinder/ (accessed on 9 September 2026)), representative sequences were aligned with MAFFT (v7.526), low-quality alignment regions were removed when necessary, and maximum-likelihood phylogenies were reconstructed with IQ-TREE (version 2.4.0) using built-in model selection and 1000 bootstrap replicates. Trees were visualized and annotated with FigTree (version v.1.4.5.). Statistical significance was defined as *p* < 0.05. Because only four animals were available in each outcome group, statistical analyses were considered exploratory and were interpreted with caution [22,23,24,25].

### 2.6. Selection of Viral Sequences for Phylogenetic Analysis

Five viral categories were selected for phylogenetic characterization based on the availability of representative assembled sequences, sufficient sequence information for phylogenetic alignment, taxonomic support from database and conserved-domain analyses, and relevance to the study objective of distinguishing potential host-associated signals from exogenous viral signals. No additional abundance or prevalence cutoff was imposed for phylogenetic selection because the study was exploratory and several candidate viral sequences were represented by partial contigs. The selected categories were therefore intended to provide representative examples of the major viral signal types identified in the dataset rather than to constitute a comprehensive phylogenetic analysis of all detected viral taxa [15,21,26,27].

## 3. Results

### 3.1. Overall Composition of the Oropharyngeal Virome in Trafficked Malayan Pangolins

**Figure 1:**
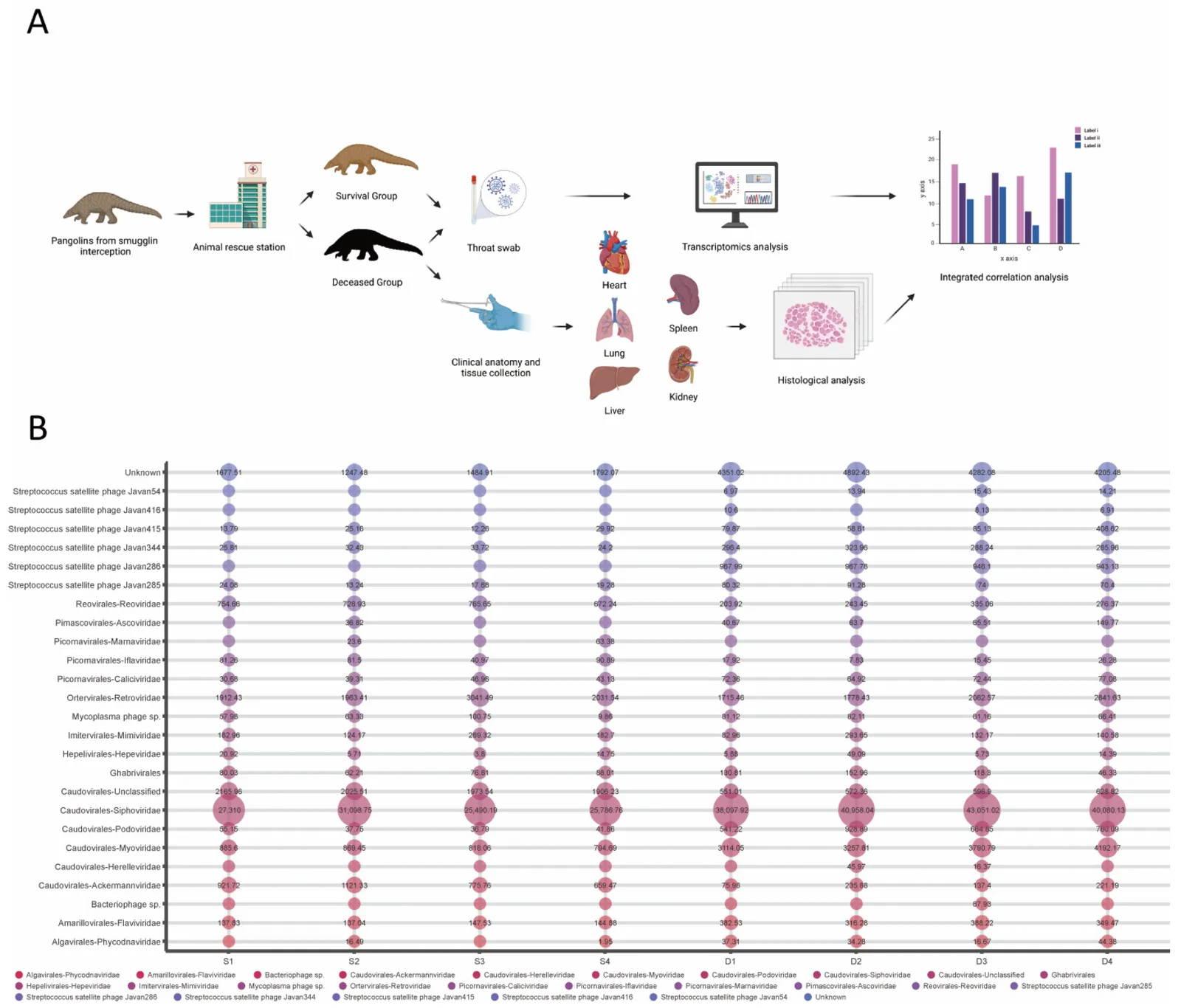
Overall composition and abundance of the oropharyngeal virome in trafficked Malayan pangolins. (**A**) Schematic overview of the study workflow. Intercepted pangolins were transferred to the rescue station and categorized into survival and deceased groups. Oropharyngeal swabs were collected for metatranscriptomic virome analysis; deceased individuals underwent necropsy and tissue sampling for pathological examination; (**B**) bubble plot showing viral taxa detected in each sample and their relative abundances. Colors represent viral taxonomic units, and bubble size indicates relative abundance (FPKM).

The eight oropharyngeal swab samples generated 330,613,818 raw reads, corresponding to 49.59 Gb of raw sequence data. After quality filtering, 318,439,724 clean reads (47.78 Gb) were retained, representing approximately 96.3% of the raw reads. Host-sequence removal yielded 313,896,204 non-host reads (47.09 Gb), corresponding to approximately 98.6% of the clean reads. The overall sequencing quality and host-read removal statistics for each sample are summarized in Table 2. The workflow of this study is shown in Figure 1A. Oropharyngeal swabs from surviving and deceased rescued pangolins were analyzed by metatranscriptomics, and tissues from deceased individuals were examined histologically.

Across all samples, the oropharyngeal virome showed a composite ecological structure (Figure 1B). Caudovirales-related taxa, satellite phages and other bacteriophage-like signals were abundant, consistent with the phage-rich nature of oral and upper gastrointestinal viromes [21,23]. Non-mammalian viral signals, including Picornavirales, Ghabrivirales, Ascoviridae, Mimiviridae and Reoviridae-related sequences, were also detected across the samples [15,21,24,25]. In parallel, lower-abundance vertebrate-associated signals, including pestivirus-like, retrovirus-like and Hepeviridae/Flaviviridae-related sequences, were detected and were consistent with previous reports of diverse vertebrate-associated viruses in pangolins [13,15,16,19,22].

The viral composition varied among individual samples (Figure 1B). Subsequent analyses therefore examined community-level diversity and the phylogenetic placement of selected viral sequences.

### 3.2. Differences in Viral Community Diversity and Composition Between Surviving and Deceased Pangolins

The difference in alpha diversity should be interpreted as a community-level signature rather than as proof that a higher number of viruses directly caused death. The observed separation in beta-diversity space indicates that viral community structure differed between the two outcome-defined groups, although the contribution of sampling time and disease progression cannot be excluded.

**Figure 2:**
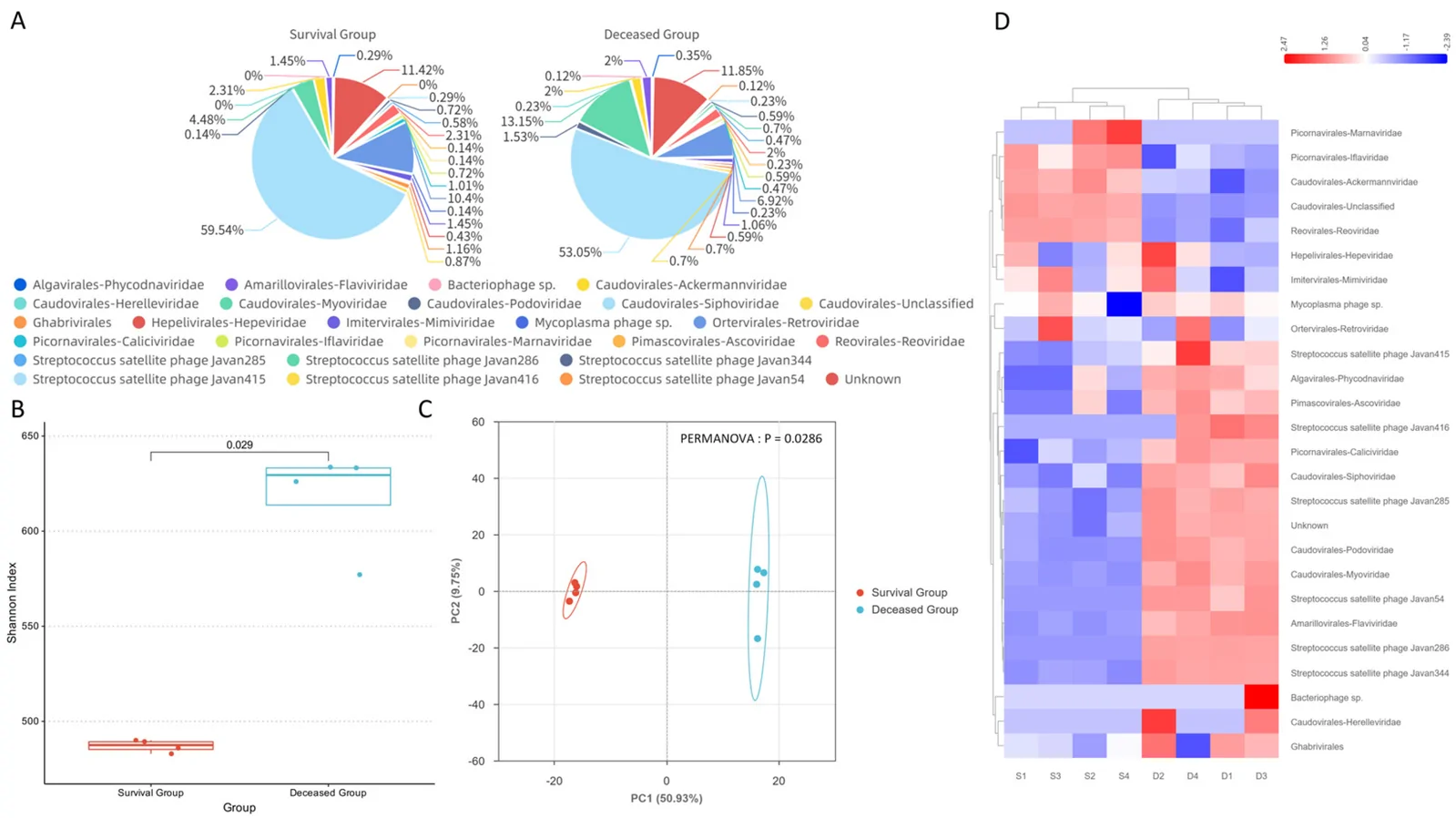
Viral community composition and diversity in surviving and deceased pangolins. (**A**) Viral composition in survival and deceased groups; (**B**) alpha diversity comparison showing significantly higher Shannon diversity in the deceased group (*p* = 0.029); (**C**) PCoA based on Bray–Curtis distances showing separation between groups (PERMANOVA, *p* = 0.0286); (**D**) heatmap of viral abundance across samples with hierarchical clustering.

Although both outcome groups retained a background dominated by phages and environment-associated signals, their overall viral compositions differed. Several viral taxa showed higher relative abundance or were detected more frequently in deceased individuals in the present dataset (Figure 2A). Similar increases in viral community complexity have been reported in diseased or stressed wild animals [21,23].

Alpha diversity analysis showed a higher Shannon diversity index in deceased pangolins than in surviving pangolins (*p* = 0.029; Figure 2B). The Shannon index reflects the diversity and evenness of the viral feature table and does not by itself indicate a higher burden of biologically relevant viral infection. Beta-diversity analysis further separated the two groups in PCoA space, and PERMANOVA indicated a difference in viral community structure between the two outcome-defined groups (*p* = 0.0286; Figure 2C). Heatmap clustering also grouped samples partly by outcome (Figure 2D). Given the small sample size of four animals per group, these findings were considered exploratory and descriptive of the present study cohort.

Based on the predefined criteria described in Section 2.5, five viral categories were selected for phylogenetic characterization: pestivirus-like, Gammaretrovirus-like, Picorna-like, Iflavirus-related, and Ghabrivirales-related sequences [15,21,26,27].

### 3.3. Phylogenetic Characterization of Selected Viral Sequences

#### 3.3.1. Pestivirus-like Sequences

Because pestiviruses are well documented in domestic and wild mammals, this lineage has higher biological relevance than many of the environmental viral signals detected in the same samples [28]. However, the use of the term Pestivirus-like remains appropriate because the present analysis relied mainly on conserved regions rather than complete isolation and experimental characterization of infectious virus.

Recent studies have reported pangolin-derived pestivirus-like viruses in deceased pangolins and in *Amblyomma javanense* ticks parasitizing pangolins [16,29]. These reports indicate that pestivirus-like sequences have been detected in pangolin-associated samples, although their biological significance and host association remain to be established.

**Figure 3:**
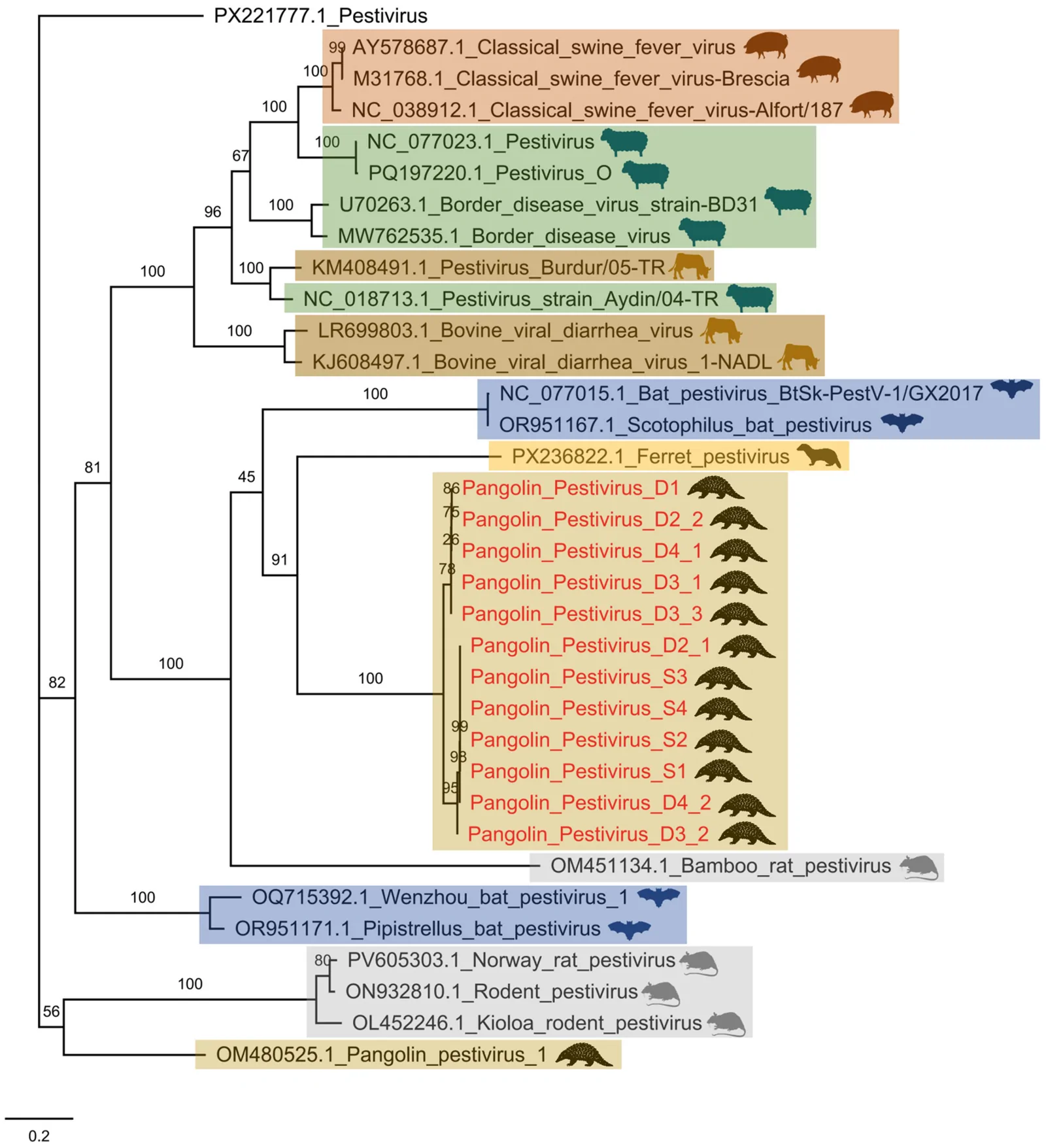
Phylogenetic analysis of pangolin pestivirus-related sequences. A maximum-likelihood tree was constructed from RdRp sequences. Sequences identified in this study are shown in red and references in black; animal icons indicate hosts. The scale bar represents 0.2 substitutions per site.

The maximum-likelihood tree based on partial RdRp sequences showed that the pestivirus-like sequences detected in this study were phylogenetically related to sequences previously reported from pangolins pestivirus sequences and other wildlife hosts (Figure 3). The detected sequences were also placed outside the major established livestock pestivirus clades. However, the relatively short contigs recovered in this study (358–838 bp) limited the resolution of phylogenetic inference, and reliable full-length nucleotide identity comparisons could not be performed. Therefore, these sequences are referred to here as pestivirus-like sequences rather than as a defined pangolin-specific or pangolin-associated lineage. The sequences were detected mainly in deceased individuals [16,28,29,30]. Because the analysis was based on oropharyngeal swabs, phylogenetic placement alone cannot establish viral replication, tissue tropism or a causal relationship with mortality. The previously reported pangolin-associated sequence OM480525.1 was relatively divergent from several sequences recovered in the present study, and some of the newly identified sequences were phylogenetically closer to pestivirus-like sequences reported from bats or ferrets. These relationships indicate that pestivirus diversity across wildlife hosts is broader than the available pangolin-associated sequences currently represent and that the evolutionary position of the present sequences remains uncertain. Accordingly, the present phylogeny should be interpreted as evidence of sequence relatedness rather than definitive evidence for a pangolin-specific lineage [31,32,33,34].

#### 3.3.2. Gammaretrovirus-like Sequences

Endogenous retroviruses (ERVs) are remnants of ancient retroviral integrations in host germline genomes and are widespread in vertebrates [35,36,37,38,39]. ERVs can be transcribed under specific biological conditions and may participate in immune regulation, inflammation or tissue homeostasis [35,37,39]. Pangolin genome analyses have identified ERV signatures from multiple retroviral genera, including Gammaretrovirus-related elements [38].

**Figure 4:**
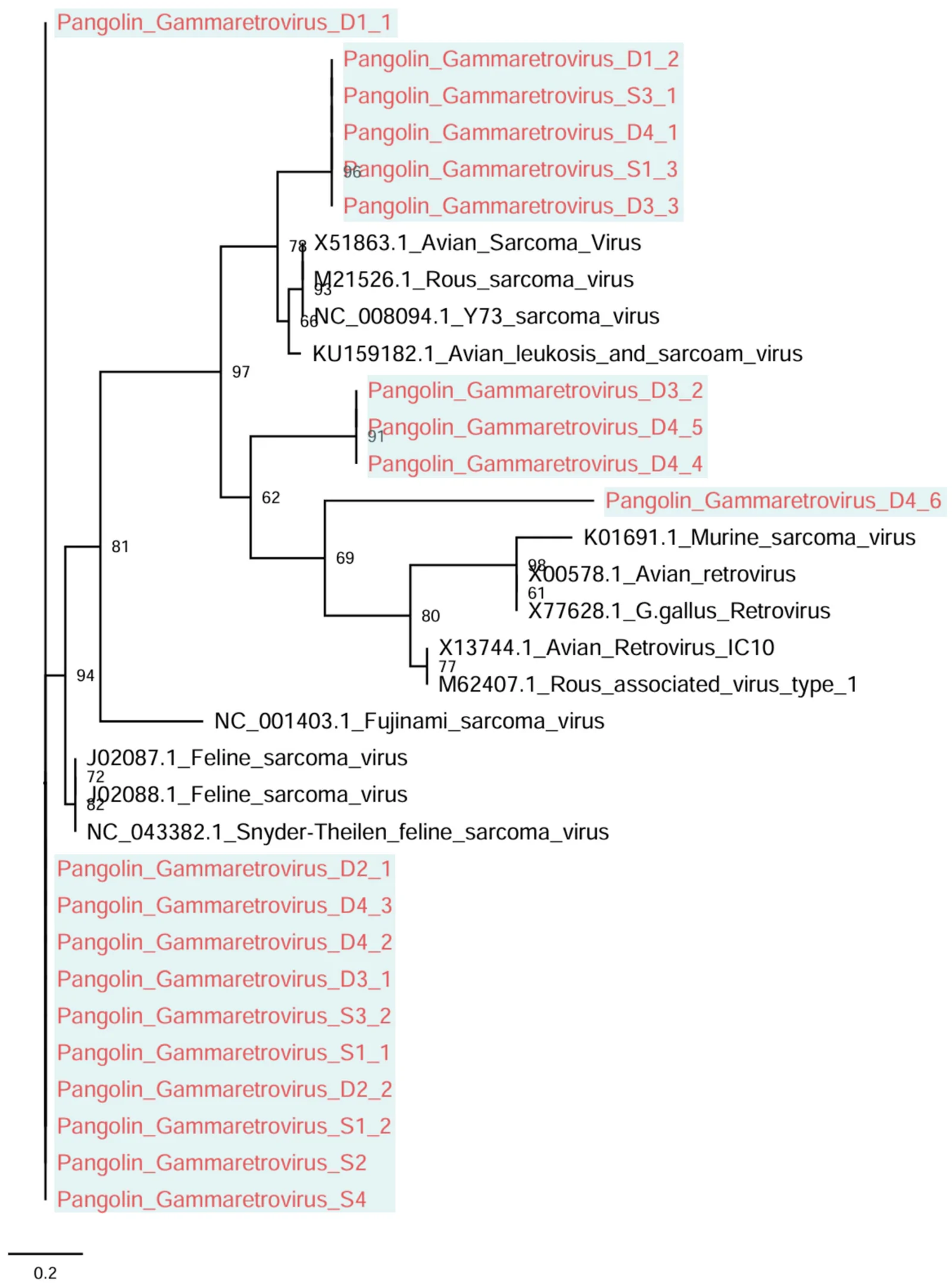
Phylogenetic analysis of Gammaretrovirus-like sequences identified in pangolins. The maximum-likelihood tree was constructed from glycoprotein-region sequences. Pangolin sequences from this study are highlighted in red and reference sequences in black. The scale bar represents 0.2 substitutions per site.

Phylogenetic analysis of glycoprotein-region sequences showed that pangolin Gammaretrovirus-like sequences were distributed across multiple lineages rather than forming a single monophyletic group (Figure 4). This pattern is compatible with heterogeneous Gammaretrovirus-like signals, including the possibility of transcripts derived from endogenous retroviral elements [35,38]. Given that oropharyngeal swabs may contain shed epithelial and inflammatory cells, detection of host genome-derived ERV transcripts is plausible. However, host-genome mapping was not performed, and endogenous origin therefore cannot be established from the present data alone.

#### 3.3.3. Picorna-like Viral Sequences

**Figure 5:**
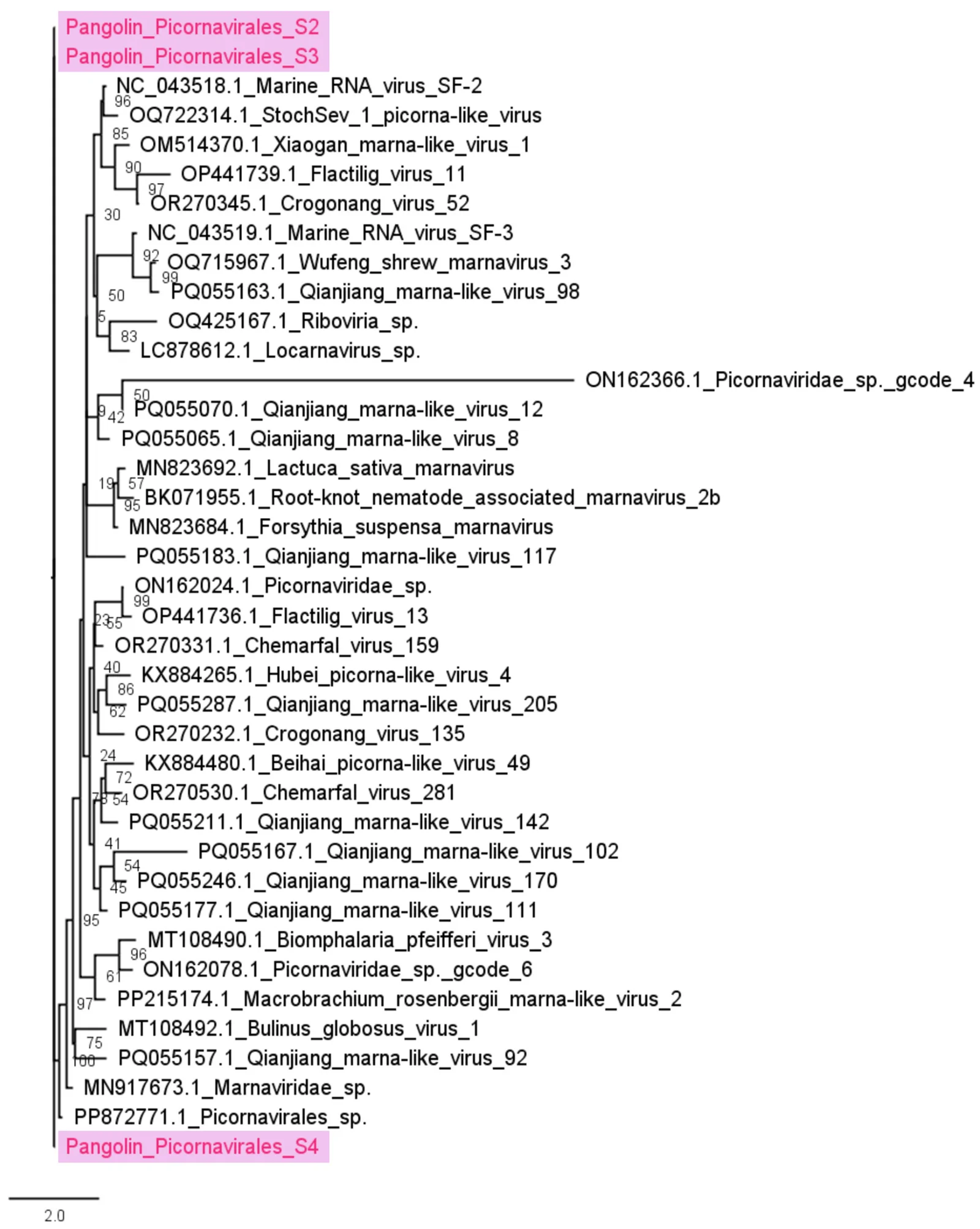
Phylogenetic analysis of picorna-like virus RdRp sequences detected in pangolins. A maximum-likelihood tree was constructed from RdRp sequences of Picornavirales-like viruses. Sequences from this study are highlighted in red and references in black. The scale bar represents 2.0 substitutions per site.

Picornavirales contains positive-sense RNA viruses with broad host ranges, including viruses infecting vertebrates, arthropods, plants and unicellular eukaryotes [26,40]. In wildlife metatranscriptomes, Picorna-like sequences may therefore represent host infection, dietary material or environmental exposure [15,21,24,25]. In the present phylogeny, the pangolin Picorna-like RdRp sequences were placed mainly among viruses of invertebrate or environmental origin rather than typical mammalian Picornaviridae (Figure 5).

Because pangolins feed primarily on ants, termites and associated material, these Picorna-like signals may be consistent with dietary- or environmental-associated viral RNA; however, their specific source could not be determined because dietary and environmental samples were not collected [15,24,25]. Their significance is therefore not that they directly explain mortality, but that they demonstrate the ecological information retained in oropharyngeal swabs. This finding supports a hierarchical interpretation of virome data in which viral phylogeny, sample type and host ecology are considered before pathogenic relevance is inferred.

### 3.4. Characteristic Pathological Lesions in Deceased Pangolins

**Figure 6:**
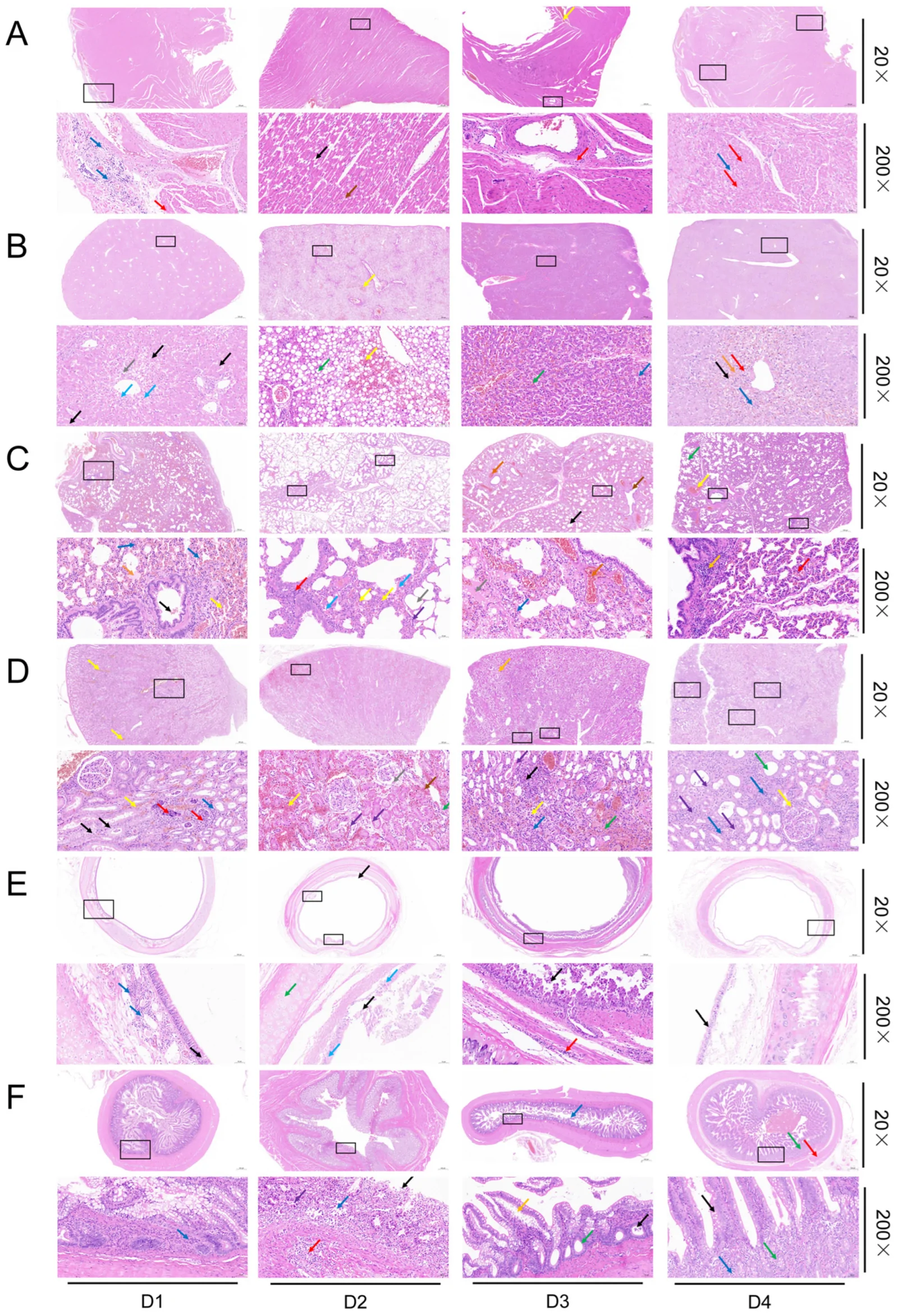
Characteristic histopathological lesions in deceased pangolins. (**A**–**F**) show H&E-stained sections of heart, liver, lung, kidney, trachea and intestine from D1–D4. For each tissue, the first row shows 20× magnification and the second row 200× magnification. Black boxes indicate high-power fields. Arrows indicate inflammatory infiltration, necrosis, hemorrhage, steatosis, fibrosis, epithelial shedding and glandular dilation; detailed arrow descriptions are provided in Appendix A.

Among the organs examined, lung and kidney lesions were particularly prominent. Histopathological examination revealed multi-organ lesions in deceased pangolins (Figure 6). Cardiac changes included myocardial fiber disruption, focal necrosis, interstitial hemorrhage and mild inflammatory infiltration. Hepatic lesions included hepatocyte edema, steatosis, hemorrhage and necrosis. Pulmonary lesions were characterized by alveolar wall thickening, vascular congestion, inflammatory infiltration and intra-alveolar exudation. Renal lesions included tubular epithelial degeneration and necrosis, interstitial hemorrhage and fibrosis. The trachea and intestine also showed mucosal epithelial injury and inflammatory changes.

These findings documented multi-organ pathological changes rather than lesions restricted to a single organ. Similar pulmonary, renal, intestinal and hemorrhagic lesions have been reported in trafficked or deceased pangolins [16,29,34,41,42,43]. In this study, the lesions co-occurred with a more complex virome in deceased individuals, but no viral in situ localization was performed. Thus, the pathological findings provide clinical and pathological context for the deceased animals, but the present data do not establish a direct relationship between the detected viral sequences and these lesions [44].

### 3.5. Viral Signatures from Pangolin Diet and Environment

**Figure 7:**
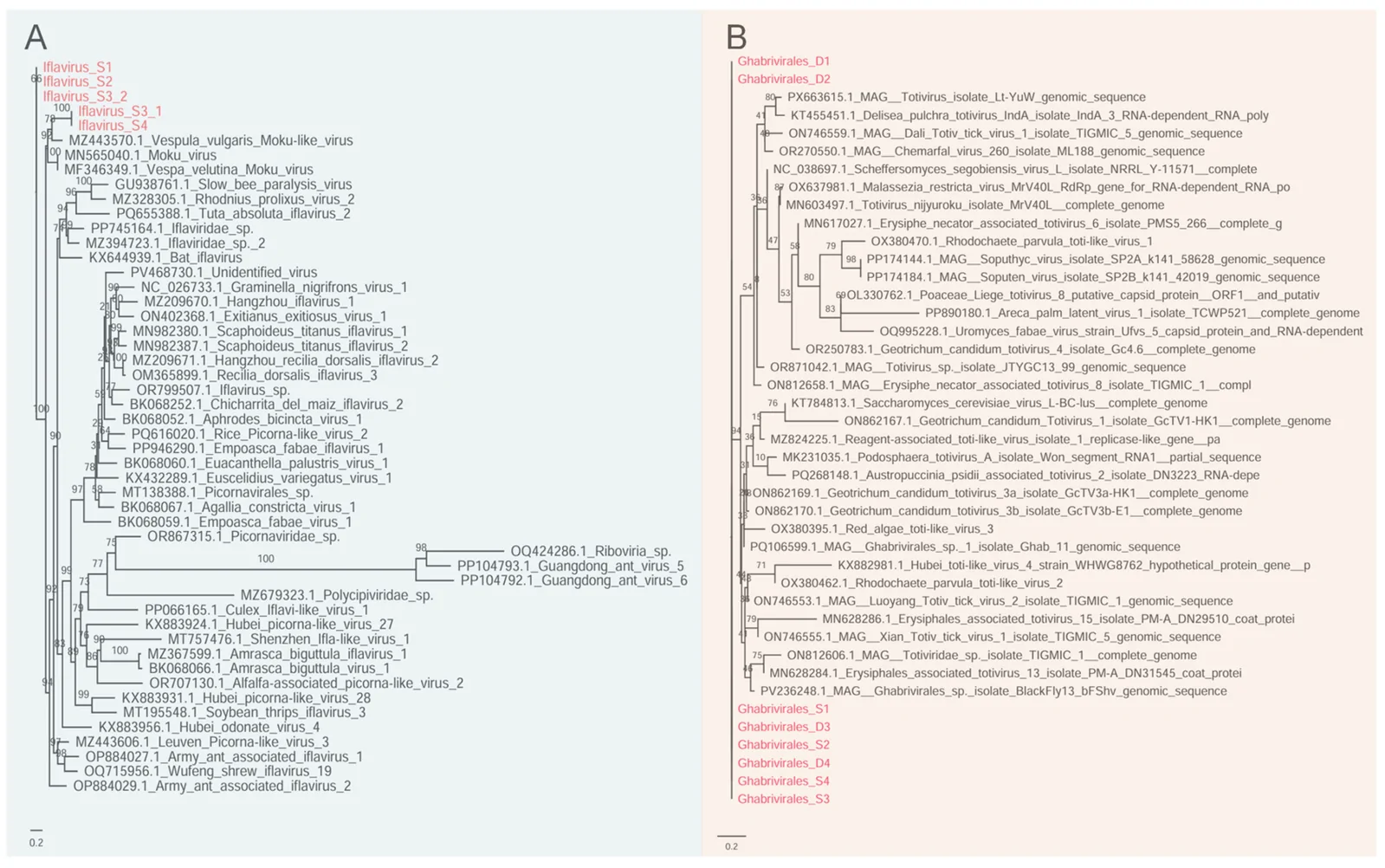
Viral signatures likely derived from pangolin diet and environment. (**A**) Maximum-likelihood tree based on partial RdRp sequences of Iflavirus-related viruses; (**B**) maximum-likelihood tree based on partial RdRp sequences of Ghabrivirales-related viruses. Pangolin sequences are highlighted in red and references in black. Scale bars represent 0.2 substitutions per site.

Exogenous viral signals from food, commensal organisms and environmental exposure are common in wildlife oropharyngeal and fecal viromes [15,21,24,25]. The Iflavirus-related sequences detected here clustered with insect-associated viruses (Figure 7A). Because Iflaviridae mainly infect arthropods, especially insects, these sequences may be consistent with viral RNA derived from dietary or environmental sources rather than stable pangolin infection [26,45]. However, the specific source of these sequences could not be determined because dietary and environmental samples were not systematically collected.

Ghabrivirales-related sequences showed affinity to dsRNA viruses associated with fungi or protists (Figure 7B). Recent ICTV taxonomic updates place many fungal-associated dsRNA viruses within revised Ghabrivirales-related groups [27,46]. Therefore, these sequences may therefore be consistent with viral signals associated with fungi, insects or other microorganisms encountered through dietary or environmental exposure. However, their specific source could not be determined in the present study. Together, Iflavirus and Ghabrivirales signals emphasize that rescued pangolin viromes record both host health and exposure history, and that exogenous signals should not be overinterpreted as host-specific pathogens.

## 4. Discussion

In this study, we observed three layers of virome signatures that together help interpret the composition of virome signals detected in rescued animals. First, the oropharyngeal virome of rescued pangolins is dominated by bacteriophages and environmental signals—a finding consistent with the composite ecological structure typical of oral and gastrointestinal viromes. Second, community-level diversity metrics differed significantly between outcome groups. Third, specific viral lineages (pestivirus-like, Gammaretrovirus-like, Picorna-like, Iflavirus and Ghabrivirales) showed distinct phylogenetic placements and distribution patterns. Below, we discuss each layer in turn, moving from the most confident findings to those requiring more caution.

The dominance of bacteriophage-related signals is important because it suggests that part of the virome reflects the bacterial component of the oropharyngeal microbiota. In contrast, the presence of arthropod-, protist- or fungus-associated viral groups is more likely to reflect ecological exposure. These two layers of information are both useful: the former may indicate local microbial community structure, whereas the latter may help reconstruct recent dietary and environmental contacts during transport and rescue.

Compared with the longer initial version, the discussion is intentionally organized around the evidence that can be supported by the present data. The main observation of this study was a difference in viral community structure between surviving and deceased pangolins within the present study cohort. The second observation concerned the occurrence and phylogenetic placement of several candidate viral sequences with different possible interpretations. Pestivirus-like sequences were considered potential host-associated candidates, although their biological significance and host association remain uncertain. Pestiviruses are positive-sense RNA viruses in the family Flaviviridae and include important livestock pathogens such as classical swine fever virus, bovine viral diarrhea virus and border disease virus [28,29,30]. These viruses can cause diverse outcomes, including subclinical infection, diarrhea, hemorrhagic disease, immunosuppression and systemic injury [28,31,32,33]. Gammaretrovirus-like sequences as potentially derived from endogenous retroviral elements, and Iflavirus/Ghabrivirales-related sequences as viral signals potentially associated with dietary or environmental exposure.

This study shows that rescued Malayan pangolins harbor heterogeneous oropharyngeal viral communities and that deceased individuals exhibited higher viral diversity, distinct community structure and multi-organ pathological lesions. These observations indicate that the virome differences occurred in the context of substantial clinical and pathological heterogeneity, but the present data do not allow the relative contributions of viral exposure, disease progression, environmental exposure or other physiological factors to be determined. This interpretation is consistent with the conditions of illegal wildlife trade, in which animals undergo capture, confinement, mixed transport, starvation, dehydration and frequent exposure to contaminated environments [3,47,48,49]. The oropharyngeal virome may contain a mixture of host-associated, dietary-associated and environmental viral signals. However, the present study does not allow the relative contributions of these sources to be distinguished. For rescue practice, the findings highlight the value of continued virome surveillance, longitudinal sampling and targeted molecular validation in wildlife rescue settings.

The pestivirus-like sequences detected here are of particular interest because they cluster with previously reported pangolin-associated pestiviruses and were mainly found in deceased pangolins [16,29,34]. This recurrence of pestivirus-like sequences in pangolin-associated datasets warrants further investigation of pangolins as potential hosts or exposed hosts. However, the present data are insufficient to determine whether these sequences represent persistent infection, transient exposure or active viral replication. The phylogenetic proximity of these sequences to previously reported pangolin-associated pestivirus-like sequences supports their consistency with a pangolin-associated pestivirus-like lineage. Establishing a natural potential host requires evidence of viral replication, persistence, tissue tropism, immune responses and repeated detection in natural populations [50,51]. Our current data, derived from oropharyngeal swabs from rescued animals, are sufficient to identify an association but not to prove that pangolins maintain or transmit this virus in the wild.

The mixed viral profile observed in this study also illustrates a broader problem in wildlife virome interpretation. Oropharyngeal swabs from trafficked animals capture host-associated viruses, phages, prey-derived viruses, microbial viruses and environment-associated viral signals, simultaneously [15,21,24,25]. In the context of wildlife transport, such complexity is expected because animals, food, ectoparasites, containers and environmental material are brought into close contact. Therefore, risk assessment should not focus only on high-profile viruses such as coronaviruses but should use broader multi-pathogen surveillance while distinguishing likely host-associated viruses from exogenous exposure signals [12,13,22].

The histopathological findings provide biological context for the virome data. Among the organs examined, lung and kidney lesions were particularly prominent, with substantial multi-organ injury documented in deceased pangolins. These lesions may have multiple possible causes, including physiological stress, circulatory disturbance, inflammation and infectious or non-infectious disease processes. Because no tissue-level viral localization or independent molecular validation was performed, the present findings do not establish a specific infectious etiology. The deceased pangolins showed pulmonary, hepatic, renal, tracheal, intestinal and cardiac lesions, a pattern compatible with systemic injury and multifactorial disease. Pestiviruses and other vertebrate viruses may contribute to immunosuppression or tissue injury in susceptible hosts [31,32,33], whereas canine parvovirus, tick-borne pathogens, parasites, dehydration and starvation have also been associated with pangolin morbidity or death [17,41,42,43]. Because this study lacked qRT-PCR validation in organs, in situ hybridization, immunohistochemistry and virus isolation, the pathological data should be interpreted as supportive rather than causal evidence.

Several limitations should be considered. First, all animals were rescued from the illegal trade rather than sampled from long-term monitored wild populations; so, the virome described here reflects rescue and trafficking contexts rather than a natural population baseline. This factor additionally caused temporal differences in sample collection timepoints between each group. The sampling times differed between the two outcome groups because animals died at different time points during the rescue period. Consequently, the two groups were not sampled at identical time points, and the observed virome differences may be influenced by captivity duration and disease progression. Given the small sample size (*n* = 8), meaningful statistical adjustment for sampling time was not feasible. Therefore, the observed differences should be interpreted cautiously as associations rather than evidence of outcome-specific viral changes. Hence, the observed group differences may still be influenced by captivity duration and disease progression, and we emphasize this as a major interpretative caveat. Second, the main sequencing samples were oropharyngeal swabs, which are useful for surveillance but cannot alone define tissue tropism or pathogenicity. Third, Gammaretrovirus-like sequences were considered potentially derived from endogenous retroviral elements based on their phylogenetic characteristics; however, without host genome mapping, their endogenous origin cannot be established, and the possibility of exogenous retroviral sequences or contamination cannot be excluded. Fourth, with only four individuals per group, the statistical power is limited, and the detected differences may partly reflect inter-individual variation rather than consistent outcome-related patterns. Finally, pangolin-derived organoid systems could provide a future route to test candidate viral tropism and epithelial responses without relying on infection experiments in endangered animals [52,53,54,55]. Overall, this study provides a framework for integrating virome profiling, phylogeny and pathology to guide pangolin rescue, pathogen surveillance and One Health risk assessment. However, whether these virome shifts precede or result from clinical deterioration requires further investigation, and causality cannot be established from this observational dataset. In addition, dietary materials and environmental samples were not systematically collected alongside the oropharyngeal swabs. Therefore, the relative contribution of dietary, environmental and host-associated viral signals could not be directly determined.

## 5. Conclusions

In conclusion, this study identified heterogeneous oropharyngeal viral communities in rescued Malayan pangolins and observed differences in viral community diversity and composition between surviving and deceased individuals. Several pestivirus-like, Gammaretrovirus-like and Picorna-like sequences were detected mainly in deceased animals, while Iflavirus- and Ghabrivirales-related sequences were more consistent with dietary-, microbial- or environmental-associated viral signals. Histopathological examination documented multi-organ lesions in deceased pangolins.

Given the small sample size, unequal sampling times between outcome groups, and the absence of independent molecular validation and tissue-level viral localization, these findings should be interpreted as exploratory associations rather than evidence of viral causality. In particular, the present study does not allow the relative contributions of host-associated, dietary-associated or environmental viral signals to rescue outcomes to be determined. Nevertheless, the study provides candidate viral signals and a framework for broader virome surveillance and future longitudinal and molecular investigations of rescued pangolins.

## Figures and Tables

**Table 1:** Information table of samples.

Sample Number	Species	Gender	Rescued Date	Clinical Sampling Date	Sampling Method	Outcome	Death Time	Grouping Number
1	Malayan pangolin	Female	2 September 2024	10 October 2024	Throat swab sampling	Survival	/	S1
2	Malayan pangolin	Female	2 September 2024	10 October 2024	Throat swab sampling	Survival	/	S2
3	Malayan pangolin	Male	2 September 2024	10 October 2024	Throat swab sampling	Survival	/	S3
4	Malayan pangolin	Female	2 September 2024	10 October 2024	Throat swab sampling	Survival	/	S4
5	Malayan pangolin	Male	2 September 2024	7 September 2024	Throat swab sampling	Death	7 September 2024	D1
6	Malayan pangolin	Female	2 September 2024	14 September 2024	Throat swab sampling	Death	14 September 2024	D2
7	Malayan pangolin	Male	2 September 2024	22 September 2024	Throat swab sampling	Death	22 September 2024	D3
8	Malayan pangolin	Female	2 September 2024	14 October 2024	Throat swab sampling	Death	14 October 2024	D4

**Table 2:** Summary of sequencing data quality and host-read removal for the eight oropharyngeal swab samples.

Sample	Raw Reads	Raw Bases (Gb)	Clean Reads	Clean Bases (Gb)	Non-Host Reads	Non-Host Bases (Gb)	Error Rate
S1	35,071,174	5.26	33,900,982	5.09	33,397,672	5.01	0.01
S2	38,231,636	5.73	36,991,974	5.55	36,454,828	5.47	0.01
S3	37,927,402	5.69	36,185,522	5.43	35,640,256	5.35	0.01
S4	39,105,574	5.87	36,918,438	5.54	36,389,714	5.46	0.01
D1	35,283,202	5.29	34,176,864	5.13	33,701,694	5.06	0.01
D2	44,172,130	6.63	42,421,376	6.36	41,805,468	6.27	0.01
D3	49,408,170	7.41	48,137,252	7.22	47,499,072	7.12	0.01
D4	51,414,530	7.71	49,707,316	7.46	49,007,500	7.35	0.01

## Data Availability

The genomic data presented in the study are openly available in [National Genomics Data Center (NGDC, CNCB)] at [accession number: CRA050246].

## Abbreviations

The following abbreviations are used in this manuscript:

IFNE	Interferon epsilon
RdRp	RNA-dependent RNA polymerase
FPKM	Fragments per kilobase of transcript per million mapped reads
PCoA	Principal coordinate analysis
ERVs	Endogenous retroviruses

## Appendix A

### Appendix A.1. Detailed Pathological Descriptions of Pangolins in Group D

#### Appendix A.1.1. A: Hematoxylin and Eosin (H&E)-Stained Heart Sections from D1 to D4

D1: Slight hyperplasia of epicardial connective tissue, accompanied by prominent lymphocytic infiltration (blue arrows). Focal myocardial necrosis is observed (red arrows), characterized by pyknotic, deeply stained nuclei and increased cytoplasmic eosinophilia.

D2: Extensive myocardial fiber rupture (black arrows) with irregular arrangement and uniform staining, along with substantial brownish-yellow pigment deposition (brown arrows). No interstitial abnormalities or significant inflammatory cell infiltration are noted.

D3: Myocardial cells exhibit clear demarcation and consistent orientation. Mild perivascular lymphocytic infiltration is present (red arrows), and large-area interstitial hemorrhage is observed (yellow arrows).

D4: The epicardial structure is intact without obvious abnormalities. Focal myocardial necrosis is detected (red arrows), featuring pyknotic, deeply stained or dissolved nuclei and increased cytoplasmic eosinophilia; occasional granulocyte infiltration is noted in the adjacent interstitium (blue arrows).

#### Appendix A.1.2. B: Hematoxylin and Eosin (H&E)-Stained Liver Sections from D1 to D4

D1: The liver capsule consists of uniformly thick dense connective tissue rich in elastic fibers. Hepatic lobule boundaries are indistinct. Centered around the central vein, prominent mild hepatocellular edema is observed (blue arrows), characterized by loose pale-staining cytoplasm. Extensive hepatocellular chromatin margination is noted (black arrows), with mild dilation of perivenous hepatic sinusoids (gray arrows). No significant necrosis or inflammatory cell infiltration is detected.

D2: The liver surface capsule is structurally intact. Widespread hepatocellular steatosis is present in the perivenous region, portal areas, and parenchyma (green arrows), with variable-sized round cytoplasmic vacuoles. Hepatic sinusoid structure and hepatocellular boundaries are obscured, with focal hemorrhagic foci scattered throughout the tissue (yellow arrows).

D3: The liver capsule is composed of uniformly thick dense connective tissue rich in elastic fibers. Central veins are located at the core of hepatic lobules, surrounded by roughly radially arranged hepatocytes and hepatic sinusoids. Mild hepatocellular edema is observed (blue arrows; swollen cells with loose pale cytoplasm), along with prominent hepatic sinusoidal dilation and congestion (green arrows).

D4: The liver capsule consists of uniformly thick dense connective tissue rich in elastic fibers. Hepatic lobule architecture is disorganized, with extensive mild hemorrhage (orange arrows; abundant erythrocytes present) and frequent brownish-yellow pigment deposition. A large number of hepatocytes are irregularly arranged, with widespread mild hydropic degeneration (black arrows; loose pale cytoplasm) and extensive necrosis (red arrows). Occasional perinecrotic lymphocytic infiltration is noted (blue arrows).

#### Appendix A.1.3. C: Hematoxylin and Eosin (H&E)-Stained Lung Sections from D1 to D4

D1: Lung parenchyma comprises branching intrapulmonary bronchi and terminal alveoli. Prominent granulocytic infiltration is observed in alveolar walls (blue arrows), with widespread moderate alveolar wall thickening, septal widening, and extensive alveolar wall capillary congestion (yellow arrows). Brownish-yellow pigment deposits are rare. Small foci of eosinophilic material are present in alveolar lumina (orange arrows), and bronchiolar hemorrhage (black arrows) is frequent, with abundant erythrocytes in lumina.

D2: The lung surface is covered by an intact smooth serosa. Mild granulocytic infiltration is noted in alveolar walls (yellow arrows), with focal mild alveolar wall thickening, septal widening, uneven alveolar sizing, and focal compensatory alveolar dilation (gray arrows). Scattered perivascular and peribronchiolar lymphocytic infiltration is present (red arrows). Focal eosinophilic fluid accumulates in alveoli (blue arrows), with occasional free macrophages and granulocytes (purple arrows).

D3: The lung surface capsule is structurally intact. Mild granulocytic infiltration is observed in alveolar walls (blue arrows), with extensive mild alveolar wall thickening, septal widening, widespread alveolar narrowing, and focal compensatory dilation (black arrows; uneven alveolar sizing). Eosinophilic fluid is present in multiple alveoli (gray arrows). Small numbers of desquamated epithelial cells and eosinophilic material are seen in bronchiolar lumina (brown arrows), with prominent vascular congestion (orange arrows).

D4: The lung surface is covered by a smooth intact serosa. Focal small-cluster lymphocytic infiltration is noted around bronchioles (orange arrows). Mild granulocytic infiltration is present in alveolar walls (red arrows), with extensive mild alveolar wall thickening and septal widening. Focal alveolar dilation (green arrows; uneven sizing) and mild vascular congestion (yellow arrows) are observed.

#### Appendix A.1.4. D: Hematoxylin and Eosin (H&E)-Stained Kidney Sections from D1 to D4

D1: The renal surface capsule consists of uniformly thick dense connective tissue. Glomeruli are evenly distributed with consistent cellularity and matrix. Prominent renal tubular epithelial degeneration is observed (black arrows), characterized by cell swelling, pale/loose cytoplasm, and small round vacuoles. Focal tubular epithelial necrosis and desquamation are noted (red arrows), with pyknotic/fragmented nuclei, accompanied by mild macrophage infiltration (blue arrows). Widespread interstitial vascular congestion is present (yellow arrows).

D2: The renal surface capsule is structurally intact. Cortical glomeruli show uniform distribution, cellularity, and matrix, with occasional eosinophilic material in Bowman’s capsule (gray arrows). Extensive tubular epithelial necrosis is detected (purple arrows), featuring pyknotic, fragmented, or dissolved nuclei; focal vacuolar degeneration of tubular epithelium is also seen (green arrows, with cytoplasmic round vacuoles). Interstitial patchy hemorrhage (yellow arrows) and prominent brownish-yellow pigment deposition (brown arrows) are observed.

D3: The renal surface capsule comprises uniformly thick dense connective tissue. Cortical glomeruli are evenly distributed, with focal Bowman’s capsule dilation (orange arrows) and eosinophilic material. Multiple small foci of tubular necrosis are present (black arrows), with pyknotic/fragmented nuclei and obscured structure, accompanied by mild lymphocytic and macrophage infiltration (yellow arrows). Necrotic cell debris and granulocytes are visible in focal tubules (blue arrows), with adjacent small-area hemorrhage (green arrows). Extensive eosinophilic material accumulates in tubules (purple arrows).

D4: The kidney exhibits extensive fibrosis, with interstitial connective tissue hyperplasia and prominent lymphocytic infiltration (blue arrows). Widespread tubular atrophy is noted (purple arrows, reduced volume and irregular shape), alongside focal mild tubular dilation (green arrows, with flattened epithelium). Glomeruli are evenly distributed, with frequent mild thickening of Bowman’s capsule walls (yellow arrows).

#### Appendix A.1.5. E: Hematoxylin and Eosin (H&E)-Stained Tracheal Sections from D1 to D4

D1: The tracheal surface is lined with pseudostratified ciliated columnar epithelium interspersed with goblet cells. Focal epithelial degeneration is noted (black arrows), characterized by cell swelling and pale loose cytoplasm. Extensive edema is present in the lamina propria and submucosa, with loose connective tissue and prominent lymphocytic and granulocytic infiltration (blue arrows). The tracheal wall is supported by dorsally open hyaline cartilage rings; the dorsal smooth muscle layer shows no significant abnormalities.

D2: The tracheal surface consists of pseudostratified ciliated columnar epithelium with goblet cells. Widespread epithelial edema is observed (blue arrows, pale/loose cytoplasm), with focal mucosal epithelial desquamation (black arrows). The underlying lamina propria and submucosa are indistinctly demarcated, with glands visible in the deep lamina propria and submucosa. Dorsally open hyaline cartilage rings (green arrows) and the dorsal smooth muscle layer show no necrosis or inflammatory cell infiltration.

D3: The trachea is supported by horseshoe-shaped hyaline cartilage rings. Tissue architecture is disorganized, with extensive mucosal epithelial necrosis and desquamation (black arrows, pyknotic/fragmented nuclei). Prominent lymphocytic infiltration is detected in the submucosa (red arrows).

D4: The tracheal surface is lined with pseudostratified ciliated columnar epithelium interspersed with goblet cells. Focal epithelial hydropic degeneration is noted (black arrows, pale/loose cytoplasm). The lamina propria and submucosa are indistinctly demarcated. Dorsally open hyaline cartilage rings and the dorsal smooth muscle layer show no significant abnormalities.

#### Appendix A.1.6. F: Hematoxylin and Eosin (H&E)-Stained Intestinal Sections from D1 to D4

D1: The mucosal layer contains abundant but irregularly arranged intestinal villi. Small foci of intestinal glands show irregular arrangement, with focal interstitial lymphocytic infiltration (blue arrows). The muscular layer exhibits regularly arranged myocytes with normal morphology.

D2: The mucosa protrudes into the intestinal lumen to form abundant folds. Multiple focal ulcers are present, with extensive mucosal epithelial desquamation (black arrows) and necrosis. Most intestinal glands in the lamina propria are structurally obscured (blue arrows), with widespread epithelial necrosis (purple arrows) and desquamation. Focal lymphocytic infiltration is noted in the submucosa (red arrows), and the muscular layer shows uneven thickness.

D3: Abundant intestinal villi (composed primarily of columnar epithelium and abundant goblet cells) are observed. Extensive villus detachment from the lamina propria is present (orange arrows), with focal villous epithelial desquamation and lamina propria exposure (blue arrows). The lamina propria contains densely packed abundant intestinal glands, with widespread glandular lumen dilation (green arrows) and focal necrotic cell debris in gland lumens (black arrows). The muscularis mucosa separates the intestinal glands from the submucosa. The submucosa consists of loose connective tissue, and the muscular layer structure is intact.

D4: The intestinal surface is lined with intestinal villi covered by morphologically normal simple columnar epithelium, interspersed with goblet cells (black arrows). The lamina propria contains abundant regularly arranged intestinal glands (green arrows), with widespread scattered interstitial lymphocytic infiltration (blue arrows). The muscularis mucosa (composed of double-layered smooth muscle) separates the intestinal crypts from the submucosa. The submucosa is connective tissue; the remaining intestinal wall (muscular layer [red arrows] and serosa) shows no significant abnormalities.
